# Patterns of Alcohol Consumption in Spanish University Alumni: Nine Years of Follow-Up

**DOI:** 10.3389/fpsyg.2017.00756

**Published:** 2017-05-15

**Authors:** Patricia Gómez, Lucía Moure-Rodríguez, Eduardo López-Caneda, Antonio Rial, Fernando Cadaveira, Francisco Caamaño-Isorna

**Affiliations:** ^1^Consumer and User Psychology Unit, Universidade de Santiago de CompostelaSantiago de Compostela, Spain; ^2^Department of Preventive Medicine and Public Health, CIBER-ESP, Faculty of Medicine, Universidade de Santiago de CompostelaSpain; ^3^Department of Clinical Psychology and Psychobiology, Universidade de Santiago de CompostelaSantiago de Compostela, Spain; ^4^Neuropsychophysiology Lab, Research Center on Psychology, School of Psychology, University of MinhoBraga, Portugal

**Keywords:** alcohol drinking in college, university students, alcohol, cluster analysis, cohort study

## Abstract

The aim of this study was to empirically identify different profiles of Spanish university alumni, based on their alcohol use over 9 years, and to further characterize them. A cohort study was carried out between 2005 and 2015 among university students (Compostela Cohort-Spain; n_2015_ = 415). Alcohol consumption was measured using the Alcohol Use Disorder Identification Test (AUDIT). A two-stage cluster analysis, based on their AUDIT total scores was carried out separately for males and females. The further characterization of every profile was based on demographic data, age at onset of alcohol use, positive alcohol-related expectancies, tobacco and cannabis use, as well as their answers to some European Addiction Severity Index items. Five different clusters were identified: *Low users* (29.2%), *Moderated users* (37.2%), *At-risk users* (14.2%), *Decreasing users* (13.2%) and *Large users* (6.2%) for females, and *Low users* (34.4%), *At-risk users* (25.6%), *High-risk users* (15.6%), *Decreasing users* (14.4%) and *Large users* (10.0%) for males. Being a cannabis user or a smoker was positively associated to those more hazardous clusters in both genders. Regarding females, significant differences in the age of onset and high positive expectancies were found. However, there were few significant differences among the groups in relation to their employment status and social relations. The results reveal the existence of different typologies of alcohol users among university alumni, with differences among males and females. Modifying positive expectancies, limiting access to alcohol at a young age, and reducing uses of other substances uses are key to promote healthier alcohol use profiles and to prevent hazardous uses.

## Introduction

Alcohol use among university students has been a subject of vast research (Mota et al., 2010; Johnston et al., 2011; White and Hingson, 2013). National and international surveys of college students usually reveal high rates of alcohol use among this age demographic (European Monitoring Center for Drugs and Drug Addiction, 2011; Substance Abuse and Mental Health Services Administration, 2013), being male students who tend to drink comparatively more than females (Courtney and Polich, 2009; Wicki et al., 2010). For instance, O'Malley and Johnston (2002) found rates around 70% of alcohol use in the last 30 days prevalence among American college students, and Moure-Rodríguez et al. (2014) found 7.8% of abstainers among college male students at 20 years old and 11.8% of abstainers among the female ones. However, most reported prevalence and consumption indicators might not be directly comparable among studies since culture-related variations and methodological differences are confounded (Wicki et al., 2010).

In addition to this, certain risk patterns of alcohol consumption, such as binge drinking are increasing among young people around the world (Jernigan, 2001). This pattern of alcohol consumption is characterized by the intake of large amounts of alcohol in a short period of time, reaching blood alcohol concentrations of 0.8 g/l or greater (National Institute on Alcohol Abuse and Alcoholism, 2016). In Spain the proportion of young people who reported having been drunk in the last 30 days increased from 25% in 2006 to 32% in 2013 (Plan Nacional Sobre Drogas, 2015). It is also worth mentioning that the literature suggests that there are aspects of the college environment that specifically tend to support alcohol drinking (O'Malley and Johnston, 2002), and that high-frequency drinking patterns that develop during university appear to persist several years post-graduation (Arria et al., 2016).

Moreover, several short-term consequences associated with an excessive alcohol consumption have been identified, such as unintentional injuries (Miller et al., 2007), having unprotected sex with casual partners (Kiene et al., 2009), drink-driving (Hingson et al., 2009), aggressions (Svensson and Landberg, 2013), or memory blackouts (Mundt et al., 2012). Likewise a growing literature have shown that some patterns of alcohol use—such as heavy or binge alcohol drinking—may lead to structural and functional anomalies in the brain as well as to deficits in several cognitive processes (Hermens et al., 2013; Jacobus and Tapert, 2013; López-Caneda et al., 2014a). Similarly, the few longitudinal studies in university students conducted to date addressing the effects of alcohol misuse in the middle/long-term report that some abnormalities in the brain function may persist or emerge if alcohol consumption is maintained (López-Caneda et al., 2012; Correas et al., 2016) whereas others may recover or brake their evolution if the binge alcohol use is ceased (Winward et al., 2014; López-Caneda et al., 2014b).

On the other hand, long-term consequences in employment status, family and social relationships during the early adulthood of university alumni have hardly been studied, mainly because of limited available longitudinal data and because much of the alcohol literature developed suggested that generally both men and women classified as problem drinkers in college tend to mature out of such behavior after college and become non-problem drinkers as adults (Perkins, 2002; Jackson and Sartor, 2016; Moure-Rodríguez et al., 2016b). Nevertheless, some authors, such as Jennison (2004) found that those with risky binge drinking style in college were either less likely to continue their education or were more likely to find work in less prestigious occupations. Likewise, several studies assessing the employment outcomes have identified long-term effects of heavy/binge drinking on employment status, showing that these risky alcohol consumption patterns were more prevalent among the unemployed (Henkel, 2011), especially in females (Berg et al., 2013).

Furthermore, many studies have observed differential effects of gender pointing to a greater vulnerability to the harmful cognitive effects of alcohol in adolescent and young females as compared to age-matched males (Caldwell et al., 2005; Nederkoorn et al., 2009; Squeglia et al., 2011). But these are not the only studies showing that the gender variable should not be only considered as a confounding factor. Multiple studies have shown important differences between females and males in prevalence of heavy episodic drinking and alcohol risky consumption, and in explicative factors of both patterns of consumption (Moure-Rodríguez et al., 2016b). Moreover, the consequences of different pattern of alcohol consumption over unsafe sex (Moure-Rodríguez et al., 2016a), car accidents (Caamaño-Isorna et al., 2017a), and alcohol related injuries (Caamaño-Isorna et al., 2017b) also have shown differences between females and males.

While heterogeneity among alcohol users has been widely recognized (Mossa et al., 2007; Leggio et al., 2009; Cortés et al., 2010), efforts to identify homogenous subpopulations of alcohol users have been focused primarily on crosssectional data (Basu et al., 2004), resulting in varied typologies with limited ability to account for high variability among alcohol users. Nevertheless, relatively little is known about longitudinal patterns of drinking behavior. In this regard, Harrington et al. (2014) identified eight distinct profiles of problematic alcohol users in an adult population, based on their daily and weekly patterns of alcohol use as well as longitudinal trajectories of drinking, while (Sunderland et al., 2014) found seven distinct profiles of Saturday night drinking behavior among young adults.

For its part, even less research has been conducted from a longitudinal point of view involving alcohol drinking trajectories in university alumni, which entail a limited understanding about how their later life could be influenced by their longitudinal pattern of drinking behavior. Johnsson et al. (2008) studied college students' drinking patterns during the first 4 years at university based on their AUDIT scores, and found four different groups: one with stable risky consumption, other one with decreasing consumption, a third group with increasing consumption, and a fourth one with stable non-risky consumption. They stated that gender influenced the trajectories, but no separate classifications were explored.

Altogether, these findings strengthen the importance to study alcohol consumption evolution at the long-term from adolescence to early-adulthood, a critical developmental transition from the cognitive point of view and the social perspective, and highlight the significance of taking into account the gender-specific patterns of alcohol drinking in order to delimit their potentially different trajectories and deleterious effects more precisely. However, longitudinal studies trying to identify different subpopulations of alcohol users in university alumni are scarce, and beyond that, to the best of our knowledge, none have been conducted separately for males and females in the Spanish context.

The aim of the present study was to empirically identify the different profiles of female and male Spanish university alumni based on their use of alcohol over 9 years, based on a cluster analysis. In addition, the clusters found were characterized in terms of antecedent variables (demographic data, age of onset of alcohol use, positive alcohol-related expectancies, and cannabis and tobacco use) and consequences, such as employment status, family and social relationships at a 9-year follow-up. Based on previous studies, we hypothesized that the effects of alcohol consumption from adolescence (aged 18–19 years) to young adulthood (from 27 to 28 years old) on the socio-economic outcomes will be stronger with persistent and increasing high alcohol consumption patterns and that these effects will be greater in women in comparison with age-matched men.

## Materials and methods

### Design, population, and sample

A cohort study was carried out to evaluate the neuropsychological and psychophysiological consequences of alcohol use among university students (Compostela Cohort-Spain). The study was carried out between November 2005 and February 2015 among students at the University of Santiago de Compostela (Spain). A cluster sampling was performed, randomly selecting at least one of the freshman year classes from the 33 university schools (a total of 53 classes). All students present in the class on the day of the survey were invited to participate in the study (*n* = 1,382). This study was approved by the Bioethics Committee of the Universidade de Santiago de Compostela. Subjects were informed both verbally and in written format, as part of the questionnaire, that participation was voluntary, anonymous, and the possibility to opt-out was available at any time. Subjects were informed that they were free to fill or refuse to fill the questionnaire. The sample used in this paper is part of this wider research project, and it is part of that used in other non-duplicate paper arising from the same longitudinal study (Moure-Rodríguez et al., 2016b).

### Data collection procedures

Participants were evaluated via a self-administered questionnaire in the classroom in November 2005 and again in November 2007. Students that provided their phone numbers were further evaluated by phone at a 4.5- and a 9.25-year follow-up. On all four occasions, alcohol consumption was measured using the Galician validated version of the Alcohol Use Disorder Identification Test (AUDIT) (Saunders et al., 1993; Varela et al., 2005). The AUDIT is a brief written screening method developed by the World Health Organization (WHO) to identify current harmful and hazardous drinking that has demonstrated reasonable psychometric properties in university students (Kokotailo et al., 2004). We decided to use the AUDIT because it is widely considered one of the best screening tests for alcohol abuse; it is transnational and it has often been used with university populations. At baseline, participants responded to additional questions about socio-demographic variables, cannabis and tobacco consumption, and positive alcohol-related expectancies. They also answered to European Addiction Severity Index (EuropASI) items about their degree, employment, family and social relationships at a 9-year follow-up.

### Definition of variables

Cannabis and tobacco consumption at 18 years old were measured with the questions “Do you consume cannabis/tobacco when you go out? Never/Sometimes/Most of the Time/Always.” The categories were recategorized to No (Never) or Yes (Sometimes, Most of the Time, Always).

Taking the number of positive and negative alcohol-related expectancies into account, a score ranging from 0 to 14 was generated (0 being the maximum of negative expectancies and 14 the maximum of positive expectancies). The scores were divided up into tertiles.

Four categories were defined for age of onset of alcohol use (After 16 years old/At 16/At 15/Before the age of 15). Alcohol use was measured through the AUDIT score at 18, 20, 22, and 27 years old—a continuous variable with values ranging from 0 to 40. The Galician validated version of the (AUDIT) (Varela et al., 2005) set the cut-off value at 5 for risky drinking, and 16 for alcohol dependence.

The EuropASI items asked about their highest degree obtained (High school-vocational training/Bachelor/Master-PhD), their longest period of employment and unemployment (Number of months), their employment pattern in the last 3 years (Employed/Student/Unemployed), their sources of financial support (Own sources-employment or unemployment subsidy-/other people's sources-family or friends-), and if their job is in line with their education (Yes/No). Likewise, they answered EuropASI questions about their current coexistence (Independent/With parents), alcohol-related problems in the home environment (Yes/No), number of close friends, and problems with parents, siblings, partner, and friends (Yes/No).

Finally, several socio-demographic variables were considered, such as place of residence (At the parents' home/Outside of the parents' home), and maternal educational level (Primary school/High school/University).

### Statistical analysis

A two-stage cluster analysis, based on their AUDIT total scores (2005, 2007, 2010 and 2015), was carried out separately for males and females. All subjects with the four aforementioned measures were included in the analysis.

Firstly, a hierarchical cluster analysis was conducted, using squared Euclidean distance as the distance measure across respondents and Ward's method for combining clusters (Ward, 1963). This method was chosen to preliminarily identify the number of clusters, since it is more powerful than other agglomerative clustering techniques that use *F*-values to maximize differences among clusters (Mojena, 1977; Hair and Black, 2002). Based on the resulting dendrograms (Milligan and Hirtle, 2003) and the change in the derived coefficients (within-cluster sum of squares) at each combination step (Burns and Burns, 2009), the five-cluster option was determined to be the optimal solution for both genders. The reliability of this solution was confirmed by entering the means of the five-cluster solution as the starting points (seeds) for an iterative k-means cluster analysis. We found 93.3% agreement in assignment of male participants to specific clusters between both methods, and 85.8% agreement in females.

To demonstrate external validity of the five types of alcohol users, a set of variables, not included in the cluster analysis but theoretically relevant to clustering variables, were used. This further characterization of every profile was based on socio-demographic variables, age at onset of alcohol use, tobacco use, cannabis use, and positive alcohol-related expectancies at the beginning of the study, as well as their answers to EuropASI items about employment, family and social relationships at the 9-year follow-up. Categorical variables were analyzed using χ^2^ analyses to determine global significance and adjusted residuals eadj (Haberman, 1973) to estimate the significance in each cell. These adjusted residuals eadj are almost independent and distributed as standard normal, so values >1.96 or < −1.96 represent a significant deviation compared to the expected value at a 95% confidence level. These residuals are useful in visualizing contingency table data, making it instantly understandable which cells are out of line with expectations, in which direction, and by how much. Continuous variables were analyzed using analysis of variance (ANOVA), and the Scheffé *post-hoc* test. Likewise effect size statistics were examined (Eta-squared and Cramer's V). All statistical analyses were conducted using the IBM SPSS Statistics v. 20.

## Results

The response rate at the 9-year follow up was 30.3% (*n* = 415; females = 325; males = 90). The characteristics of the initial sample and the follow-up samples in both genders were analyzed in relation to maternal educational level, residence, age of onset of use of alcohol, positive expectations about alcohol, AUDIT total score, cannabis consumption and tobacco consumption. There were no significant differences in relation to any of these variables, neither among females nor males, as summarized in Tables 1, 2, respectively.

**Table 1:** **Characteristics of female initial sample and follow-up samples**.

	**Percentage or mean (95%CI)**				***p*-value**
	**Initial (2005) (18-19 years old) *n* = 992**	**2-year follow-up (2007) (20–21 years old) *n* = 669**	**4-year follow-up (2010) (22–23 years old) *n* = 461**	**9-year follow-up (2015) (27–28 years old) *n* = 325**	
**MATERNAL EDUCATIONAL LEVEL**					
Primary school	41.8 (38.4–45.3)	44.2 (40.1–48.4)	43.1 (38.3–48.3)	45.7 (40.1–51.8)	
High school	33.6 (30.2–37.1)	30.5 (26.4–34.7)	30.6 (25.8–35.8)	28.1 (22.5–34.2)	
University	24.6 (21.2–28.1)	25.3 (21.3–29.6)	26.3 (21.4–31.4)	26.2 (20.7–32.4)	0.642
**RESIDENCE**					
In parental home	24.7 (22.1–27.5)	22.9 (19.7–26.1)	22.2 (18.5–26.0)	20.9 (16.5–25.1)	
Away from the parental home	75.3 (72.6–78.0)	77.1 (74.0–80.3)	77.8 (74.1–81.6)	79.1 (74.9–83.5)	0.720
**POSITIVE EXPECTATIONS ABOUT ALCOHOL**					
Low	37.1 (33.4–40.9)	37.5 (33.2–42.1)	36.5 (31.4–42.0)	37.9 (31.7–44.3)	
Medium	34.0 (30.3–37.8)	32.6 (28.3–37.3)	34.6 (29.4–40.1)	34.8 (28.6–41.2)	
High	28.9 (25.2–32.7)	29.9 (25.5–34.5)	28.9(23.7–34.4)	27.2 (21.0–33.6)	0.999
**AGE OF ONSET OF USE OF ALCOHOL**					
After 16 years old	19.0 (16.5–21.8)	17.9 (14.9–21.3)	16.5 (13.0–20.5)	14.5 (10.5 – 19.2)	
At 16 years old	38.9 (35.6–42.2)	38.1 (34.1–42.2)	36.8 (32.0 – 41.7)	36.6 (30.9 – 42.6)	
At 15 years old	25.6 (22.7–28.7)	25.9 (22.3–29.6)	26.5 (22.2–31.1)	28.3 (23.0 – 34.0)	
Before age of 15 years	16.5 (14.0–19.7)	18.1 (15.0–21.5)	20.3 (16.4–24.5)	20.7 (16.0–25.9)	0.438
AUDIT: Total (mean)	5.4 (5.2–5.7)	5.6 (5.1–5.8)	5.6 (5.2–6.0)	5.3 (4.9–5.8)	0.884
Cannabis consumption	18.6 (16.2–21.1)	19.0 (15.9–22.0)	20.6 (16.8–24.4)	18.8 (14.4–23.2)	0.942
Tobacco consumption	31.0 (28.1–34.0)	31.5 (27.9–35.1)	34.3 (29.8–38.7)	32.9 (27.7–38.2)	0.786

**Table 2:** **Characteristics of male initial sample and follow-up samples**.

	**Percentage or mean (95%CI)**				***p*-value**
	**Initial (2005) (18–19 years old) *n* = 371**	**2-year follow-up (2007) (20–21 years old) *n* = 206**	**4-year follow-up (2010) (22–23 years old) *n* = 139**	**9-year follow-up (2015) (27–28 years old) *n* = 90**	
**MATERNAL EDUCATIONAL LEVEL**					
Primary school	32.0 (26.5–37.8)	35.8 (28.4–43.3)	41.6 (32.8–50.8)	41.6 (31.5–53.5)	
High school	27.6 (22.1–33.3)	27.4 (19.9–34.9)	25.5 (16.8–34.7)	27.0 (16.8–38.9)	
University	40.3 (34.8–46.0)	36.8 (29.3–44.3)	32.8 (24.1–42.0)	31.5 (21.3–43.4)	0.449
**RESIDENCE**					
In the parental home	29.7(25.1–34.5)	27.8 (21.9–34.1)	28.8 (21.6–36.4)	28.9 (20.0–38.3)	
Away from the parental home	70.3 (65.7–75.1)	72.2 (66.3–78.5)	71.2 (64.0–78.9)	71.7 (62.2–80.5)	0.949
**POSITIVE EXPECTATIONS ABOUT ALCOHOL**					
Low	29.7 (23.7–36.0)	33.0 (25.1–41.0)	34.2 (25.0–44.3)	31.6 (20.3–43.7)	
Medium	38.0 (32.0–44.4)	30.7 (22.9–38.8)	31.7 (22.5–41.8)	30.4 (19.0–42.5)	
High	32.3 (26.3–38.7)	36.3 (28.5–44.4)	34.2 (25.0–44.3)	38.0 (26.6–50.0)	0.705
**AGE OF ONSET OF ALCOHOL USE**					
After 16 years old	18.1 (12.5–24.1)	16.8 (9.2–24.7)	15.5 (6.9–25.5)	18.2 (7.8–30.3)	
At 16 years old	36.9 (31.2–42.8)	41.0 (33.5–49.0)	44.0 (35.3–54.0)	48.1 (37.7–60.1)	
At 15 years old	21.6 (15.9–27.5)	20.2 (12.7–28.2)	21.6 (12.9–1.6)	20.8 (10.4–32.8)	
Before age of 15 years	23.4 (17.8–29.4)	22.0 (14.4–30.0)	19.0 (10.3–9.0)	13.0 (2.6–25.1)	0.381
AUDIT: Total (mean)	7.8 (7.2–8.4)	7.4 (6.6–8.2)	7.3 (6.4–8.2)	7.1 (6.0–8.2)	0.784
Cannabis consumption	27.0 (22.3–31.6)	27.7 (21.3–34.0)	25.9 (18.3–33.5)	24.4 (15.0–33.9)	0.885
Tobacco consumption	27.5 (22.8–32.2)	21.8 (16.0–27.7)	23.0 (15.7–30.4)	24.4 (15.0–33.9)	0.636

### Cluster solution

**Figure 1:**
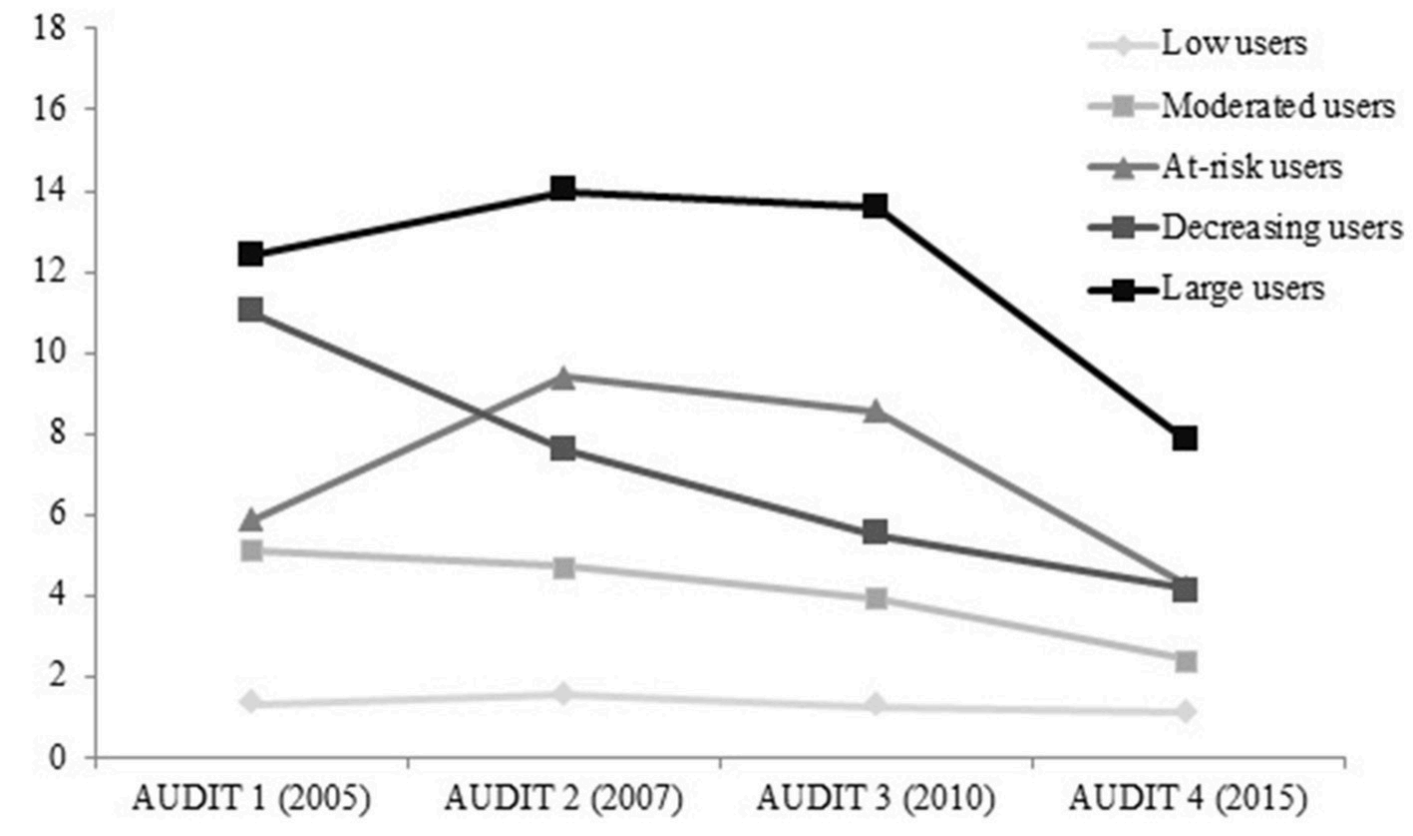
**Pattern of alcohol consumption by cluster (Females) based on the AUDIT total score (mean)**.

Table 3 shows that the clustering solution provided statistically significant differences among the five clusters on every clustering variable. In the case of females (Figure 1), the group 1, labeled as Low alcohol users, had the lowest mean scores over time (never above 1.58. At the other extreme, the Large users (group 5) had the highest scores during this 9-year follow up. Between these two clusters, three other groups emerged with different patterns of use. The group 2, the Moderated users, had low scores over time (from 5.13 to 2.42); the At-risk users (group 3) got mean scores in the range from 4.22 to 9.39; and the group 4, labeled as Decreasing users, progressively reduced their scores over time (from 11.00 to 4.16). For every cluster, the last score (AUDIT 4-2015) was the lowest one.

**Table 3:** **Descriptive statistics of clustering variables by group**.

**Gender**	**Cluster**	**Label**	**n (%)**	**Clustering variables**			
				**AUDIT 1 (2005)**	**AUDIT 2 (2007)**	**AUDIT 3 (2010)**	**AUDIT 4 (2015)**
Female	1	Low users	95 (29.2)	1.33^2,3,4,5^	1.58^2,3,4,5^	1.27^2,3,4,5^	1.13^2,3,4,5^
	2	Moderated users	121 (37.2)	5.13^1,4,5^	4.73^1,3,4,5^	3.93^1,3,4,5^	2.42^1,3,4,5^
	3	At-risk users	46 (14.2)	5.85^1,4,5^	9.39^1,2,4,5^	8.54^1,2,4,5^	4.22^1,2,5^
	4	Decreasing users	43 (13.2)	11.00^1,2,3^	7.60^1,2,3,5^	5.51^1,2,3,5^	4.16^1,2,5^
	5	Large users	20 (6.2)	12.40^1,2,3^	14.00^1,2,3,4^	13.60^1,2,3,4^	7.80^1,2,3,4^
TOTAL			325	5.34	5.42	4.61	2.86
Male	1	Low users	31 (34.4)	2.32^2,3,4,5^	3.10^2,3,4,5^	2.94^2,3,4,5^	1.97^2,3,4,5^
	2	At-risk users	23 (25.6)	5.35^1,3,4,5^	7.70^1,3,5^	8.30^1^	5.87^1^
	3	High-risk users	14 (15.6)	9.64^1,2,5^	11.57^1,2,4,5^	10.07^1,4^	6.00^1^
	4	Decreasing users	13 (14.4)	11.92^1,2,5^	7.85^1,3,5^	6.38^1,3,5^	4.62^1^
	5	Large users	9 (10.0)	16.44^1,2,3,4^	16.22^1,2,3,4^	11.44^1,4^	6.33^1^
TOTAL			90	7.03	7.59	6.77	4.41

1,2,3,4,5 *Significantly different clusters (Scheffé test; α = 0.05)*.

**Figure 2:**
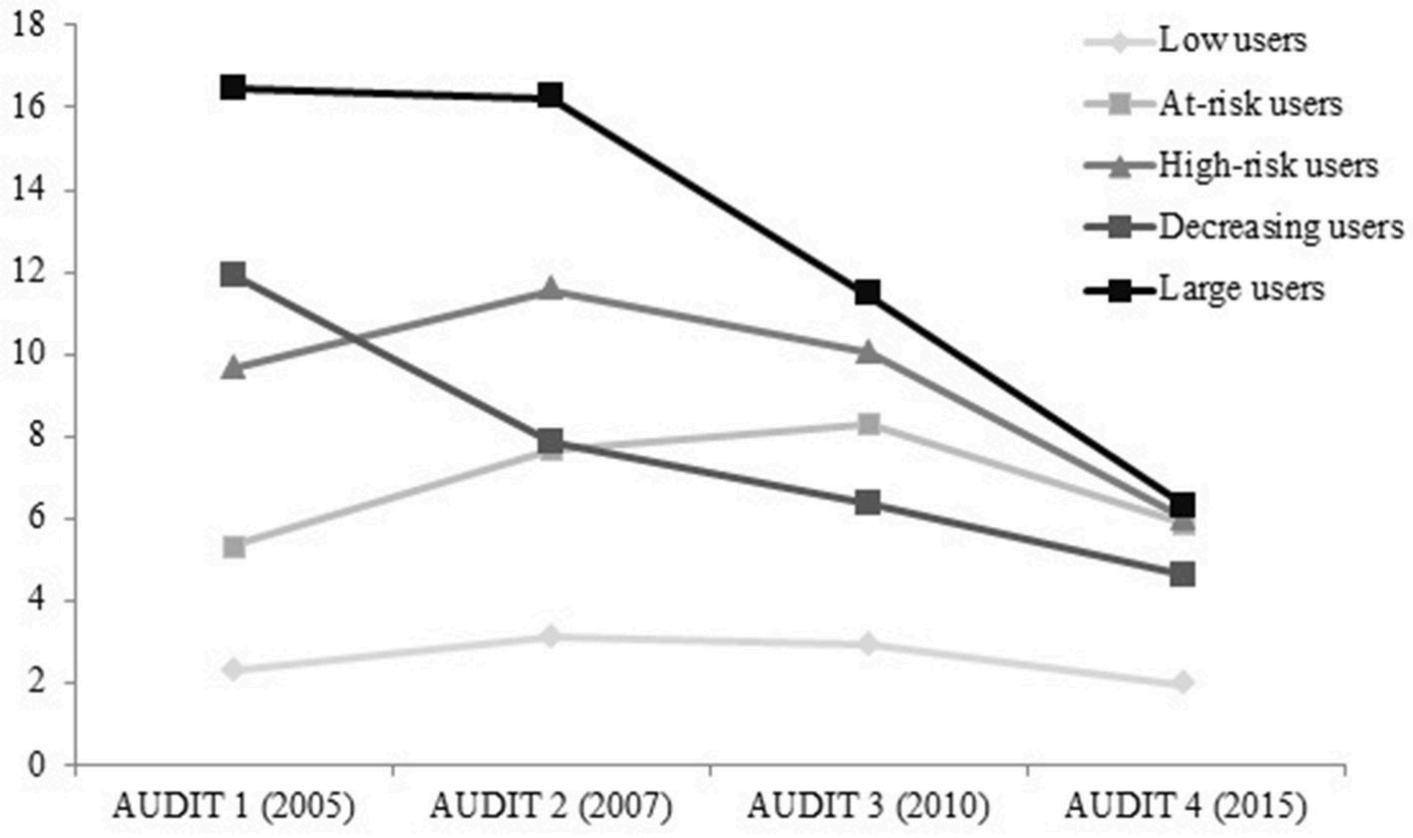
**Pattern of alcohol consumption by cluster (Males) based on the AUDIT total score (mean)**.

In the case of males (Figure 2), the Low users group (cluster 1) got the lowest scores over time (never above 3.10). At the other end, the group 5, labeled as Large users, got the highest mean scores over time. Moreover, there were three more different clusters: the At-risk users (group 2), with mean scores in the range from 5.35 to 8.30; the High-risk users (group 3), with scores never below 6; and the Decreasing users (group 4), whose mean scores decreased from 11.92 to 4.62. Among males, the last score was also the lowest one.

### Antecedent variables

Table 4 shows the differences found in the antecedent variables among the five female-clusters. With regard to the age of onset of use, statistically significant differences were found. The *Low users* had a significantly lower percentage of females who start drinking before or at 15 (17.4%), and a significantly higher percentage who start after 16 (55.1%). In the case of *Large users*, a significantly higher percentage of them started drinking before 15 (45.0%). The positive alcohol-related expectancies also exhibited significant differences between groups. As such, while the *Low users* had a significantly higher percentage of women with low positive expectancies, the *At-risk, Decreasing* and *Large users* displayed a significantly higher percentage of women with high positive expectancies. Furthermore, the *Low users* has a significantly lower percentage of females who are cannabis users (1.1%) or smokers (2.1%), while the *Large users* group includes comparatively more cannabis users (70.0%) and smokers (70.0%) than expected.

**Table 4:** **Descriptive statistics of antecedent variables by cluster (Females)**.

	**Test statistic**	**Low users (%)**	**Moderated users (%)**	**At-risk users (%)**	**Decreasing users (%)**	**Large users (%)**	**TOTAL (%)**
**MATERNAL EDUCATIONAL LEVEL**							
Primary school	χ^2^ = 7.640	49.5	50.0	43.5	34.9	30.0	45.7
High school	*p* = 0.469;	28.4	26.7	30.4	27.9	30.0	28.1
University	*V* = 0.109	22.1	23.3	26.1	37.2	40.0	26.2
**RESIDENCE**							
At the parents' home	χ^2^ = 6.064; *p* = 0.194; *V* = 0.137	26.3	23.1	17.4	11.6	10.0	20.9
Outside the parents' home		73.7	76.9	82.6	88.4	90.0	79.1
**AGE OF ONSET OF ALCOHOL USE**							
Before 15	χ^2^ = 88.199	8.7^−^	20.8	18.2	21.4	45.0^+^	19.3
At 15	*p* < 0.001	8.7^−^	26.7	34.1	47.6^+^	25.0	26.4
At16	*V* = 0.316	27.5	41.7^+^	38.6	23.8	25.0	34.2
After 16 years old		55.1^+^	10.8^−^	9.1^−^	7.1^−^	5.0	20.0
**POSITIVE EXPECTANCIES**							
Low	χ^2^ = 60.025	66.3^+^	5.3	27.5	5.6^−^	6.7^−^	37.9
Medium	*p* < 0.001	19.3^−^	41.4	32.5	52.8^+^	33.3	34.8
High	*V* = 0.322	14.5^−^	23.3	40.0^+^	41.7^+^	60.0^+^	27.2
Cannabis users	χ^2^ = 59.467; *p* < 0.001; *V* = 0.428	1.1^−^	17.4	26.1	30.2^+^	70.0^+^	18.8
Smokers	χ^2^ = 63.814; *p* < 0.001; *V* = 0.443	2.1^−^	42.1^+^	45.7^+^	44.2	70.0^+^	32.9

+,− *Significant (positive or negative) associations between the cluster and the category of variable (standardized residuals; α = 0.05)*.

Table 5 shows the differences found in the antecedent variables among the five male-clusters. In this case, statistically significant differences were found in terms of being a cannabis user or a smoker. This is similar to what has been noted earlier in relation to females, the *Low users* had a significantly lower percentage of members who were cannabis users (3.2%) or smokers (6.5%), while the *Large users* group included comparatively more cannabis users (66.7%) and smokers (55.6%) than expected.

**Table 5:** **Descriptive statistics of antecedent variables by cluster (Males)**.

	**Test statistic**	**Low users (%)**	**At-risk users (%)**	**High-risk users (%)**	**Decreasing users (%)**	**Large users (%)**	**TOTAL (%)**
**MATERNAL EDUCATIONAL LEVEL**							
Primary school	χ^2^ = 6.837	46.7	52.2	28.6	30.8	33.3	41.6
High school	*p* = 0.554	33.3	21.7	21.4	23.1	33.3	27.0
University	*V* = 0.196	20.0	26.1	50.0	46.2	33.3	31.5
**RESIDENCE**							
At the parents' home	χ^2^ = 5.353; *p* = 0.253; *V* = 0.244	38.7	30.4	28.6	23.1	0.0^−^	28.9
Outside the parents' home		61.3	69.6	71.4	76.9	100.0^+^	71.1
**AGE OF ONSET OF ALCOHOL USE**							
Before 15	χ^2^ = 14.942	8.0	4.5	7.7	23.1	33.3^+^	12.2
At 15	*p* = 0.245	16.0	27.3	15.4	23.1	11.1	19.5
At16	*V* = 0.246	36.0	45.5	53.8	46.2	55.6	45.1
After 16 years old		40.0^+^	22.7	23.1	7.7	0.0	23.2
**POSITIVE EXPECTANCIES**							
Low	χ^2^ = 11.220	48.1^+^	33.3	21.4	18.2	0.0	31.6
Medium	*p* = 0.190	25.9^−^	23.8	50.0	27.3	33.3	30.4
High	*V* = 0.266	25.9	42.9	28.6	54.5	66.7	38.0
Cannabis users	χ^2^ = 23.145; *p* < 0.001; *V* = 0.507	3.2^−^	26.1	14.3	53.8^+^	66.7^+^	24.4
Smokers	χ^2^ = 11.008; *p* < 0.05; *V* = 0.350	6.5^−^	30.4	28.6	30.8	55.6^+^	24.4

+,− *Significant (positive or negative) associations between the cluster and the category of variable (standardized residuals; α = 0.05)*.

### Employment status, family, and social relationships

Table 6 shows the differences between the female-clusters and their employment status, and family and social relationships. In relation to their education, there was a significantly higher percentage of females who reached a Master's degree or a PhD level among the Large users. Moreover, the number of close friends was found to be significantly different between Low users (4.24) and Large users (6.25). On the other hand, the At-risk users group had a significantly higher percentage of members who have problems in their home environment. In relation to having serious problems with their partner, Low users were negatively significant associated (1.1%), while At-risk (10.9%) and Large users (15.0%) were positively significant associated.

**Table 6:** **Descriptive statistics of employment status, family and social relationships at the 9-year follow-up by cluster (Females)**.

	**Test statistic**	**Low users**	**Moderated users**	**At-risk users**	**Decreasing users**	**Large users**	**TOTAL**
**HIGHEST DEGREE OBTAINED**							
High school/ Vocational training	χ^2^ = 12.237; *p* = 0.141	5.3%	2.5%	2.2%	0.0%	0.0%	2.8%
Bachelor	*V* = 0.137	84.2%	82.6%	87.0%	83.7%	65.0%^−^	82.8%
Master/PhD		10.5%	14.9%	10.9%	16.3%	35.0%^+^	14.5%
Longest period of employment (months)	*F =* 0.364; *p* = 0.834; η^2^ = 0.005	25.53	24.36	25.22	24.84	19.65	24.60
Longest period of unemployment (months)	*F =* 1.217; *p* = 0.304; η^2^ = 0.015	8.32	9.07	6.52	5.40	7.25	7.90
**PATTERN OF EMPLOYMENT IN THE LAST 3 YEARS**							
Employed	χ^2^ = 2.302	70.5%	70.2%	80.4%	86.0%^+^	65.0%	73.5%
Student	*p* = 0.138	22.1%	14.9%	15.2%	9.3%	25.0%	16.9%
Unemployed	*V* = 0.138	7.4%	14.9%^+^	4.3%	4.7%	10.0%	9.5%
**SOURCE OF FINANCIAL SUPPORT**							
Own sources (employment or unemployment subsidy)	χ^2^ = 4.892; *p* = 0.299; *V* = 0.123	68.4%	75.2%	80.4%	83.7%	70.0%	74.8%
Other people's sources (family or friends)		31.6%	24.8%	19.6%	16.3%	30.0%	25.2%
Job in line with their education	χ^2^ = 6.499; *p* = 0.165; *V* = 0.150	68.4%	73.1%	52.4%^−^	65.9%	58.8%	66.9%
No. of close friends	F = 4.227; *p* < 0.05; η^2^ = 0.050	4.24^5^	4.61	5.33	4.95	6.25^1^	4.75
**CURRENT COEXISTENCE**	χ^2^ = 0.225						
Independent	*p* = 0.994	65.3%	67.8%	65.2%	65.1%	65.0%	66.2%
With parents	*V* = 0.026	34.7%	32.2%	34.8%	34.9%	35.0%	33.8%
Alcohol-related problems in the home environment	χ^2^ = 20.287; *p* < .001; *V* = 0.269	1.3%	0.0%	10.8%^+^	0.0%	0.0%	1.8%
**SERIOUS PROBLEMS**							
With their parents	χ^2^ = 2.110; *p* = 0.716; *V* = 0.081	5.3%	4.1%	8.7%	7.0%	10.0%	5.8%
With their siblings	χ^2^ = 2.150; *p* = 0.708; *V* = 0.081	2.1%	3.3%	2.2%	0.0%	0.0%	2.2%
With their partner	χ^2^ = 12.302; *p* < 0.05; *V* = 0.195	1.1%^−^	4.1%	10.9%^+^	2.3%	15.0%^+^	4.6%
With their friends	χ^2^ = 3.823; *p* = 0.430; *V* = 0.108	3.2%	5.0%	10.9%	4.7%	5.0%	5.2%

+,− *Significant (positive or negative) associations between the cluster and the category of variable (standardized residuals; α = 0.05)*.

1,2,3,4,5 *Significantly different clusters (Scheffé test; α = 0.05)*.

In the case of males (Table 7), no difference in their employment status, and family and social relationships were found to be significant.

**Table 7:** **Descriptive statistics of employment status, family and social relationships at the 9-year follow-up by Cluster (Males)**.

	**Test statistic**	**Low users**	**At-risk users**	**High-risk users**	**Decreasing users**	**Large users**	**TOTAL**
**HIGHEST DEGREE OBTAINED**							
High school/ Vocational training	χ^2^ = 7.987	3.2%	0.0%	14.3%	15.4%	11.1%	6.7%
Bachelor	*p* = 0.435	77.4%	73.9%	71.4%	76.9%	55.6%	73.3%
Master/PhD	*V* = 0.211	19.4%	26.1%	14.3%	7.7%	33.3%	20.0%
Longest period of unemployment (months)	F = 0.876; *p* = 0.482; η^2^ = 0.040	8.61	5.26	9.14	12.23	6.33	8.13
**PATTERN OF EMPLOYMENT IN THE LAST 3 YEARS**							
Employed	χ^2^ = 8.929	67.7%	87.0%	78.6%	76.9%	66.7%	75.6%
Student	*p* = 0.348	25.8%	8.7%	7.1%	7.7%	33.3%	16.7%
Unemployed	*V* = 0.223	6.5%	4.3%	14.3%	15.4%	0.0%	7.8%
**SOURCE OF FINANCIAL SUPPORT**							
Own sources (employment or unemployment subsidy)	χ^2^ = 2.866; *p* = 0.580; *V* = 0.178	77.4%	87.0%	85.7%	69.2%	66.7%	78.9%
Other people's sources (family or friends)		22.6%	13.0%	14.3%	30.8%	33.3%	21.1%
Job in line with their education	χ^2^ = 2.041; *p* = 0.728; *V* = 0.155	61.3%	77.3%	71.4%	58.3%	66.7%	67.1%
No. of close friends	F = 1.497; *p* = 0.210; η^2^ = 0.066	5.13	4.61	6.43	5.54	5.11	5.26
**CURRENT COEXISTENCE**	χ^2^ = 1.854						
Independent	*p* = 0.763	61.3%	52.2%	57.1%	61.5%	77.8%	60.0%
With parents	*V* = 0.144	38.7%	47.8%	42.9%	38.5%	22.2%	40.0%
Alcohol-related problems in the home environment		0.0%	0.0%	0.0%	0.0%	0.0%	0.0%
**SERIOUS PROBLEMS**							
With their parents	χ^2^ = 5.808; *p* = 0.214; *V* = 0.254	16.1%^+^	4.3%	0.0%	0.0%	11.1%	7.8%
With their siblings	χ^2^ = 5.907; *p* = 0.206; *V* = 0.256	9.7%^+^	0.0%	0.0%	0.0%	0.0%	3.3%
With their partner	χ^2^ = 2.726; *p* = 0.605; *V* = 0.174	3.2%	0.0%	7.1%	0.0%	0.0%	2.2%
With their friends	χ^2^ = 1.374; *p* = 0.849; *V* = 0.124	6.5%	8.7%	7.1%	0.0%	11.1%	6.7%

+,− *Significant (positive or negative) associations between the cluster and the category of variable (standardized residuals; α = 0.05)*.

## Discussion

The major finding of this study was the characterization of five different clusters of university alumni based on their pattern of alcohol use at a 9-year follow-up, separately for females (Low users, Moderated users, At-risk users, Decreasing users and Large users) and males (Low users, At-risk users, High-risk users, Decreasing users and Large users). These groups are similar to those found by Johnsson et al. (2008) based on college students' drinking patterns during the first 4 years at university: one group with stable non-risky consumption (similar to our Low and Moderate users group), another with increasing consumption (similar to our At-Risk and High-risk users), a third one with decreasing consumption (our Decreasing users) and a last one with stable risky consumption (our Large users). The main differences between these two classifications could come from the gender division of our sample and the longer period of our follow-up, that allow us to refine—in terms of gender and alcohol consumption typology—and divide more precisely their group with stable non-risky consumption into Low users and Moderate users in the case of females, as well as their group with increasing consumption into At-risk users and High-risk users in the case of males.

Our results show that the clusters are different for females and males. This fact highlights the relevance of analyzing the data separately, and it is related to a repeated finding in the literature on gender difference in alcohol use: women drink less alcohol than men, something that also occurs among college students (Ham and Hope, 2003).

Regarding the evolution of alcohol consumption over the years, although the five clusters for each gender are very different among them, there is a generalized reduction of the AUDIT scores at the 9-year assessment for every profile, which suggests a common “mature out” of such behavior in the late 20s (Moure-Rodríguez et al., 2016b). This commonality in developmental trajectories has been found previously not only about alcohol use and heavy drinking, but also about smoking, and marijuana use (Chen and Jacobson, 2012).

Moreover, the differences among these five groups in terms of antecedents were examined. In the case of females, significant differences in relation to the age of onset of use were revealed. Our findings are in line with those that point out that the earlier age of onset, the heavier use over the years (Pitkänen et al., 2005; Mota et al., 2010). Likewise, the high positive alcohol-expectancies are found to be related to those more hazardous profiles (At-risk, Decreasing, and Large users), while low positive alcohol-expectancies are associated to the Low users. This is a consistent finding with previous researches (Griffin et al., 2000; Young et al., 2006; Caamaño-Isorna et al., 2008), and highlights the relevance of the positive early expectancies about alcohol use in present and future uses.

Being a cannabis user or a smoker is positively associated to those more hazardous clusters and negatively associated to the Low users, for both females and males. This is a finding in agreement with previous researches (American Academy of Pediatrics. Committee on Substance Abuse, 2001; Hingson et al., 2004), which highlights the harmful role of the polysubstance use.

At this point, it is of utmost importance to note that the main prevention efforts should be set in the adolescence period, because the codes of behavior acquired at that time tend to be maintained in adulthood (Grant et al., 2005). Our findings suggest that the prevention strategies should take into account that modifying positive expectancies, and reducing other substances uses are key to promote healthier alcohol use profiles and to prevent hazardous uses. For instance, the value-based education and life skill training approach has already shown its effectiveness in preventing risky behaviors, such as alcohol or substance abuse in adolescents (European Monitoring Centre for Drugs and Drug Addiction, 2008). In addition, regulatory development and legal control are necessary to limit access to alcohol at young ages.

In relation to their social relationships at the 9-year follow-up, the number of close friends was significantly higher among female Large users than female Low users. This could be related to the fact that a high percentage of Large users have reached a Master or PhD, in the sense that college attendance provides an environmental context affording greater opportunities for drinking (Carter et al., 2010) and keeping in touch with friends, and may prolong the sense of being in-between childhood and the responsibilities of adulthood (Merrill and Carey, 2016), compared to those who have already joined the labor market.

On the other hand, the females from the At-risk and Large users groups are positively associated to serious problems with their partner. This finding is in line with previous studies reporting that a persistent drinking trajectory is associated with being separated, divorced or never married (Schulenberg et al., 1996; Hicks et al., 2010). However, in the case of males, there is no difference among clusters in having serious problems with their partner, a gender difference in line with some previous researches (Cranford et al., 2011, 2015) that could be explained by the fact that alcohol consumption is part of the male gender role (Iwamoto and Smiler, 2013).

Finally, there were no significant differences among clusters in most of the analyzed consequences, in the case of either females or males. However, it might be thought that differences in employment and social situations will become greater and significant later on their lives. The continuation of this research project will allow us to confirm or refute this hypothesis in the future.

There are three possible limitations in our study. (1) Selection bias and non-representativeness, because of the loss of subjects in the follow-up, especially in the case of the small sample of males. However, the statistical analysis found no significant differences between the initial and follow-up samples in relevant variables neither in males nor in females. Nevertheless, future studies might confirm the subgroups found among males with a larger sample. (2) Since the question about expectancies is not specifically validated, expectancies may have not been correctly measured. (3) This study relied on self-report measures, so it is impossible to know if participants have underreported or over-reported their uses, if their responses were biased by gender stereotypes activation, or even by inconsistent personal feelings or memories related to their age. Nonetheless, the AUDIT questionnaire has been internationally validated in adolescents and young adults, and self-report of alcohol and other drug use has been demonstrated to be usually reliable or even better than other approaches to detect substance use (Babor et al., 1989; Winters et al., 1990).

The major strength of the study is the 9-year follow-up of Spanish university alumni with longitudinal measures of drinking, as well as the use of a cluster analysis technique to females and males separately. Our results reveal the existence of dissimilar typologies of alcohol users in Spanish university alumni, which were in turn different for males and females. There were few significant differences among the groups in relation to their employment status and social relations at the 9-year follow up. For its part, the differences among the groups found in terms of antecedents suggest that the prevention strategies should take into account that modifying positive expectancies, limiting access to alcohol at young ages, and reducing other substances uses are key to promote healthier alcohol use profiles and to prevent harmful uses.

## Author contributions

FC-I, FC designed the study. LM, EL collected the data. PG, FC-I, AR, LM analyzed and interpreted data. PG wrote the first version of the manuscript. All authors collaborated on writing the final article, have approved the final version for publication and guarantee the accuracy or integrity of this work in all its aspects.

## Funding

This work was supported by a grant from the Plan Nacional sobre Drogas (Spain) (2005/PN014) and from Fondo de Investigación Sanitaria (Spain) (PI15/00165). Eduardo López-Caneda was supported by the SFRH/BPD/109750/2015 Postdoctoral Fellowship of the Portuguese Foundation for Science and Technology as well as by the Psychology Research Centre (UID/PSI/01662/2013), co-financed by FEDER through COMPETE2020 under the PT2020 Partnership Agreement (POCI-01-0145-FEDER-007653).

### Conflict of interest statement

The authors declare that the research was conducted in the absence of any commercial or financial relationships that could be construed as a potential conflict of interest.
